# Increased Tea Saponin Content Influences the Diversity and Function of Plantation Soil Microbiomes

**DOI:** 10.1128/spectrum.02324-21

**Published:** 2022-01-12

**Authors:** Shouke Zhang, Junqia Kong, Longfei Chen, Kai Guo, Xudong Zhou

**Affiliations:** a State Key Laboratory of Subtropical Silviculture, Zhejiang A&F University, Hangzhou, Zhejiang, People’s Republic of China; b School of Forestry and Biotechnology, Zhejiang A&F University, Hangzhou, Zhejiang, People’s Republic of China; c Northwest Institute of Eco-Environment and Resources, Chinese Academy of Sciences, Lanzhou, Gansu, People’s Republic of China; Lerner Research Institute

**Keywords:** plant secondary metabolites, soil microbiome, tea saponin, plantation ages, soil physicochemical properties, core function

## Abstract

Plant secondary metabolites (PSMs) can affect the structures and functions of soil microbiomes. However, the core bacteria associated with PSMs, and their corresponding functions have not been explored extensively. In this study, soil physicochemical properties, tea saponin (TS) contents, microbial community compositions, and microbial community functions of different-age Camellia oleifera plantation soils from representative regions were analyzed. We evaluated the effects of plantation age increase on PSM accumulation, and the subsequent consequences on the structures and functions of soil microbiomes. Plantation ages increase positively correlated with accumulated TS contents, negative effects on soil physicochemical properties, and soil microbiome structures and functions. Clearly, the core functions of soil microbiomes transitioned to those associated with PSM metabolisms, while microbial pathways involved in cellulose degradation were inhibited. Our study systematically explored the influences of PSMs on soil microbiomes via the investigation of key bacterial populations and their functional pathways. With the increase in planting years, increased TS content simplified soil microbiome diversity, inhibited the degradation of organic matter, and enriched the genes related to the degradation of TS. These findings significantly advance our understanding on PSMs-microbiome interactions and could provide fundamental and important data for sustainable management of *Camellia* plantations.

**IMPORTANCE** Plant secondary metabolites (PSMs) contained in plant litter will be released into soil with the decomposition process, which will affect the diversity and function of soil microbiomes. The response of soil microbiomes to PSMs in terms of diversity and function can provide an important theoretical basis for plantations to put forward rational soil ecological management measures. The effects of planting years on PSM content, soil physicochemical properties, microbial diversity, and function, as well as the interaction between each index in *Camellia oleifera* plantation soil are still unclear. We found that, with planting years increased, the accumulation of tea saponin (TS) led to drastic changes in the diversity and function of soil microbiomes, which hindered the decomposition of organic matter and enriched many genes related to PSM degradation. We first found that soil bacteria, represented by Acinetobacter, were significantly associated with TS degradation. Our results provide important data for proposing rational soil management measures for pure forest plantations.

## INTRODUCTION

Artificial management leads to specific plants becoming the dominant species within a particular area and their litter can significantly impact soil characteristics, including soil microbial composition and function (1, 2). Plant litter decomposition is a complex, long-term process (2–4). In addition to organic matter and nutrients, plant secondary metabolites (PSMs) that are recalcitrant to degradation are also released into soil (3). PSMs play important roles in ecosystem processes such as plant succession and litter decomposition by regulating interactions between plants and soil microorganisms (3). Healthy and stable soil microbiomes with high diversity can promote soil decomposition, providing an appropriate environment ideal for plant growth. However, most PSMs also have bacteriostatic effects which lead to the degradation of critical soil physicochemical properties (5).

Many studies indicated that microbiomes are influenced by numerous types of molecules, including coumarins, glucosinolates, benzoxazines, and triterpenoids (1, 6–9). These PSMs can exert a wide spectrum of effects upon individual microbial strains or groups, particularly when they function as toxins (1). Consequently, microbiome responses to PSMs primarily result in changes to microbial structures (10–14) and their functions accordingly (1, 13, 14). These changes include the circulation of soil carbon, nitrogen, phosphorus, and other substances that directly affect plant growth and development and are closely related to plant health (4). Thus, decreased plant growth occurs mainly due to continuous planting of a single species over many years in a large area, and to the degradation of circulation (4, 15, 16). For example, dominant plants have more significant effects on soil abiotic and biotic properties (15, 16) which, in turn, influence litter decomposition via changes to decomposer communities. Previous studies have solely evaluated the relationship between soil microbiome changes, soil physicochemical properties, and plant health (1, 13, 14). The core bacteria associated with PSMs, and their functions, thus deserve further investigation.

*Camellia oleifera* is considered an economically important woody edible oil crop and over 4 million ha of plantation have been established in the mountainous areas of southern China (17). This industry plays a critical role in alleviating poverty, and its direct economic output is worth approximately 6.5 billion USD annually (18). However, *Camellia* plantation management with limited knowledge-based human intervention has led to weakened, older *Camellia* plantations that are much less productive and are hardly profitable (17, 19–21). Most studies have attributed the results to the poor resistance of older *C. oleifera* to diseases and insects (17, 19–21), although soil compaction and high densities have also been investigated. *Camellia* plantations are generally established on hillsides, and insufficient management has led to less soil compaction due to excessive fertilization (17, 21). Therefore, it is possible that other factors influence the deterioration of conditions in older *C. oleifera* plantations. *Camellia oleifera* tissues are rich in the triterpenoid saponin (10 to 20% concentration), which is a tea saponin composed of sugar chains and triterpenoids in addition to steroids or steroid alkaloids linked by carbon-oxygen bonds; this saponin is an important PSM involved in disease and pest resistance (17, 21, 22). As planting years increase, *C. oleifera* litters are bound to accumulate and considerable amounts of PSMs, mainly tea saponin, may also decompose into the soil. Consequently, we propose the hypothesis that tea saponin affects the composition and function of soil microbiomes in older *C. oleifera* plantations, thereby influencing overall soil ecological functions.

To explore this hypothesis, soil samples were collected from three major oil tea-producing areas in China. In each area, soil samples from different planting years were collected. The relationships between soil physicochemical properties, plantation ages, and tea saponin accumulation levels were analyzed. In addition, 16S rRNA gene high-throughput sequencing and soil metagenomic sequencing were performed to analyze the effects of planting period and tea saponin content on soil microbiome structures and functions, respectively. Our results provide important insights into the reconstruction of less-productive *C. oleifera* plantations, and a framework for analysis and management improvement.

## RESULTS

### Soil physicochemical properties in different-age *C. oleifera* plantations.

**FIG 1:**
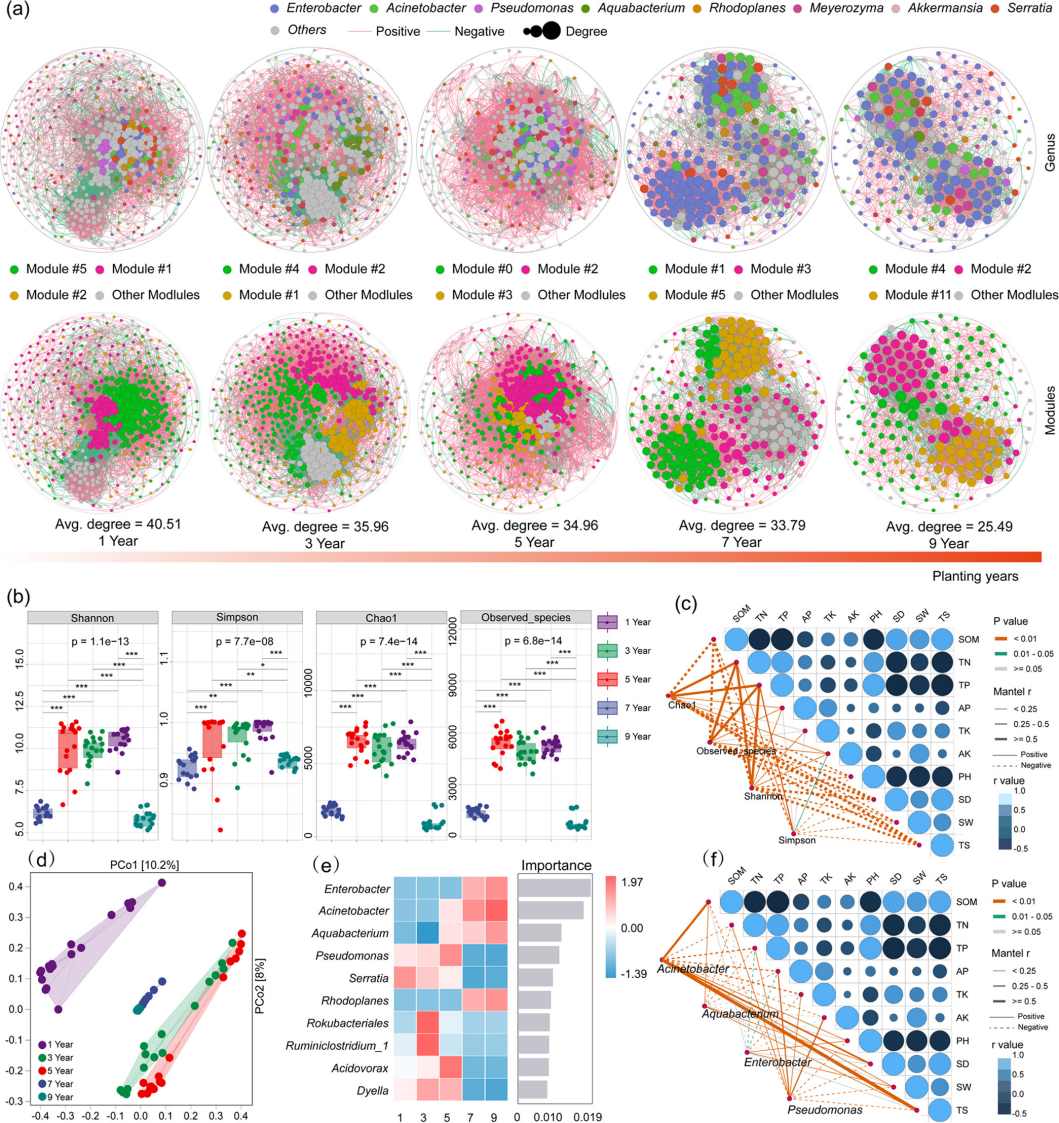
Effects of *C. oleifera* plantation ages on soil microbiome structures and diversities. (a) Network of soil microbial populations among *C. oleifera* plantations ageing from 1 to 9 years. Red lines indicate positive correlations and green lines for negative correlations. The area of the node is proportional to node degree, calculated from correlations of abundances for each ASV. Only correlations with an *r *of >0.6 or <−0.6 and a *P* value of <0.05 were included in the network. (b) Alpha diversity values for soil microbial communities among plantations of various ages. (c) Correlations between soil physicochemical parameters and alpha diversity index values. (d) PCoA analysis of soil community compositional variation based on Bray-Curtis distances. (e) Random forest classification analysis of dominant bacteria in plantations of different ages. (f) Correlations between the abundances of dominant bacteria identified by random forest classification as discriminatory for sample groups with soil physicochemical properties.

Soil physicochemical properties clearly differed among *C. oleifera* soils from plantations of various ages. Specifically, soil organic matter (SOM), soil density (SD), and tea saponin (TS) were significantly and positively correlated with *Camellia* planting years (*R*_2_ > 0.8, *P* < 0.05) (see Fig. S1a, i, k in the supplemental material). Total nitrogen (TN), total phosphorus (TP), and pH were also significantly and negatively correlated with increased planting years (*R*^2^ > 0.75, *P* < 0.05) (Fig. S1b, c, h). A positive correlation was observed between SW and planting years (*R*^2^ = 0.59, *P* < 0.05) (Fig. S1j). AP, TK, and AK correlated moderately. However, AP and TK initially increased with planting years and then decreased, while AK content fluctuated among groups (Fig. S1e, f, g). The accumulation of tea saponin with increased planting years was significantly and positively correlated with SD and moisture content (SW) (Fig. 1c). In contrast, TN, TP, and pH were negatively correlated with tea saponin accumulation.

### Effects of *C. oleifera* plantation age on soil microbiome structures and diversities.

A co-occurrence network analysis indicated that the species composition of the soil microbiomes significantly changed with increasing planting years. Obviously dominant flora were not present in the soil microbiomes of 1- to 5-year-old plantations, and no significant differences were observed in the proportions of each genus which represented between 2% and 7% of the overall communities (Fig. 1a). They began to appear in the 7- to 9-year-old *C. oleifera* plantation soils. Specifically, Enterobacter (50.41%), Acinetobacter (11.79%), and *Serratia* (4.07%) became dominant in soils after the seventh year of *C. oleifera* cultivation. Acinetobacter abundance further increased in 9-year-old plantations (14.36%) (Fig. 1a). Bacterial richness and network complexity gradually decreased from the first year (with an average degree of 40.51) to the fifth year (average degree of 34.96) and then to the ninth year (average degree of 25.49), with the last representing the lowest richness and network complexity (Fig. 1a, Table 1). The number of ‘hub nodes’ (nodes with high degree values, >60; and closeness centrality, >0.3) in the network gradually decreased with increasing planting years (Fig. 1a; Table 1).

**TABLE 1:** Bacterial co-occurrence network characteristics of *C. oleifera* planting years

Niche/yr	Node	Positive edge	Negative edge	Avg. degree	Modularity^*a*^	Avg. clustering coefficient^*b*^	Avg. path distance^*c*^
1	524	7234	3379	40.508	1.169	0.591	2.951
3	524	6773	2660	35.966	1.173	0.54	2.779
5	462	4501	1523	34.694	1.0968	0.625	2.513
7	246	3005	1152	33.797	1.062	0.664	2.429
9	195	1720	765	25.487	1.319	0.682	2.321

a Degree of nodes tending to differentiate into different network modules.

b Degree of nodes tending to cluster together.

c Network path distance is the length of the shortest path between two nodes within the network.

Thus, *C. oleifera* tree age had a strong effect on bacterial alpha diversity (i.e., based on Shannon, Simpson, Chao1, and observed richness indices) and network complexity (i.e., a higher average degree, representing a greater network complexity) (Fig. 1b and c). Among soil physicochemical properties, soil organic matter (SOM), pH, SD, SW, and TS were negatively correlated with the four alpha diversity indices (Fig. 1c). PCoA analyses of soil microbiomes were conducted using Bray-Curtis distances and indicated that soil microbiome samples from across planting years could be clustered into three groups. The first group included soil samples taken after the first year of planting, while the second group included samples from 3- to 5-year-old plantations and the third group included samples from 7- to 9-year-old plantations (Fig. 1d and Table 2). Random forest classification was used to identify discriminatory taxa for soils of different-age plantations, and revealed that Enterobacter, Acinetobacter, *Aquabacterium*, and *Rhodoplanes* were significantly enriched in 7- to 9-year plantation soil microbiomes (Fig. 1e).

**TABLE 2:** Intragroup and intergroup Adonis analysis based on Bray-Curtis distance

Plantation age (yr)	Group	*df* ^*a*^	Sums of sqs	Mean sqs	F model	*R* ^2^	*P* value
1	Intra-	2	1.86	0.93	7.17	0.49	0.001
	Inter-	15	1.95	0.13		0.51	
	Total	17	3.81			1.00	
3	Intra-	2	1.66	0.83	5.53	0.42	0.001
	Inter-	15	2.25	0.15		0.58	
	Total	17	3.92			1.00	
5	Intra-	2	3.11	1.56	12.32	0.62	0.001
	Inter-	15	1.90	0.13		0.38	
	Total	17	5.01			1.00	
7	Intra-	2	0.18	0.09	5.00	0.60	0.001
	Inter-	15	0.27	0.02		0.40	
	Total	17	0.45			1.00	
9	Intra-	2	0.11	0.06	9.63	0.56	0.001
	Inter-	15	0.09	0.01		0.44	
	Total	17	0.20			1.00	

a Degrees of freedom.

**FIG 2:**
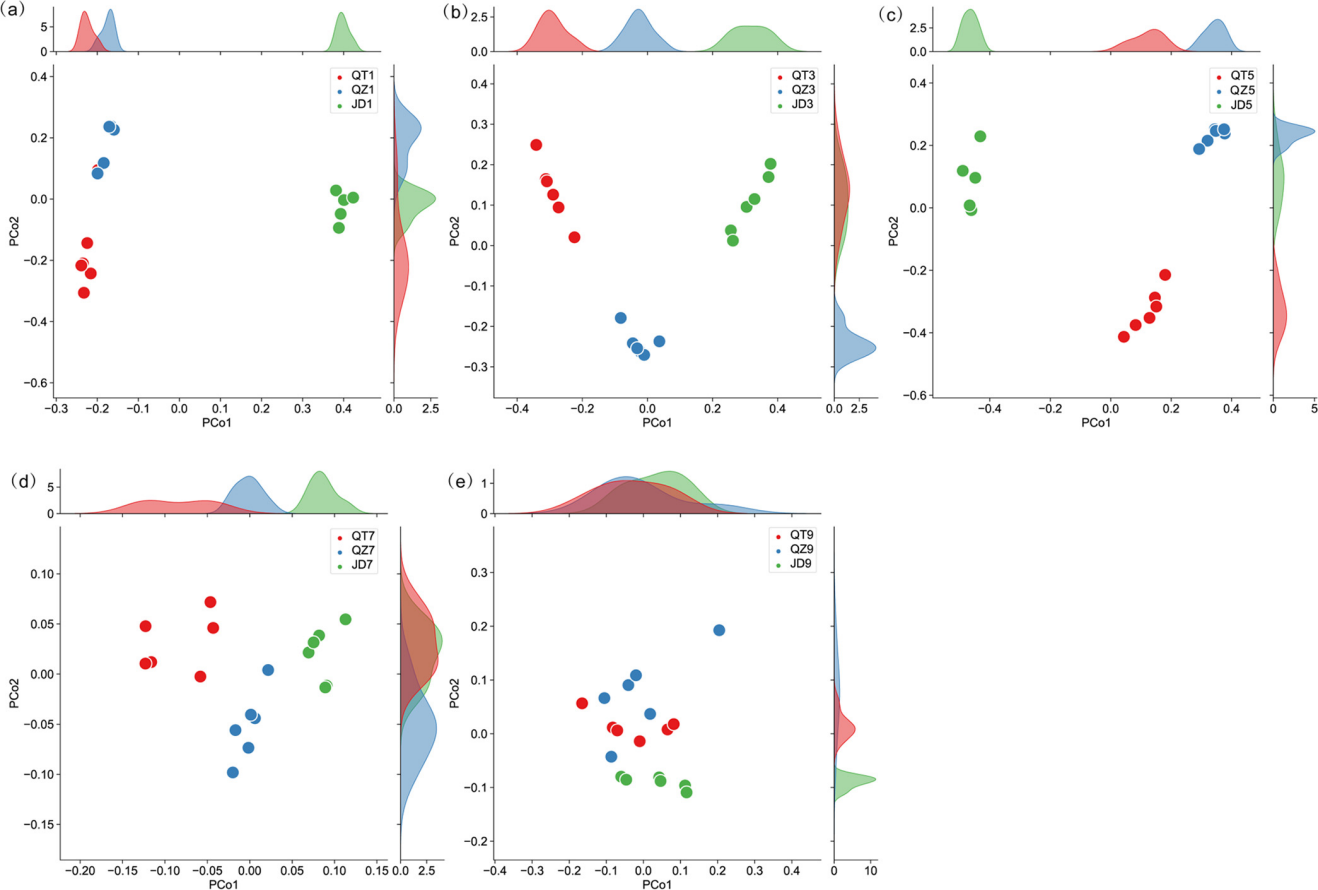
Effects of *C. oleifera* plantation ages on soil microbial community compositional variation among different sampling sites. (a to e) Comparison of similarity between samples of plantation soils from different regions based on Bray-Curtis distances.

Correlation analysis of the relative abundances of these four genera and of soil physicochemical indices demonstrated that the accumulation of tea saponin was significantly and positively correlated with increased relative abundance of Acinetobacter (Fig. 1f). Increased age of an *C. oleifera* plantation was associated with more similar soil microbiome compositions among different regions (Fig. 2 and Table 2). As indicated above, soil microbiome samples could be clustered into three groups (QT, QZ, and JD) according to bacterial community similarity (Fig. 2 and Table 2). Furthermore, soil microbiome community composition became homogenous over time. For these groups, the soils from 1-, 3-, and 5-year-old C. oleifera plantations showed intragroup differences, but the soils from 7- to 9-year-old *C. oleifera* plantations exhibited the highest intragroup similarities (Fig. 2 and Table 2), in agreement with the results of the co-occurrence network analysis (Fig. 1a).

### Effects of *C. oleifera* plantation age on soil microbiome functions.

**FIG 3:**
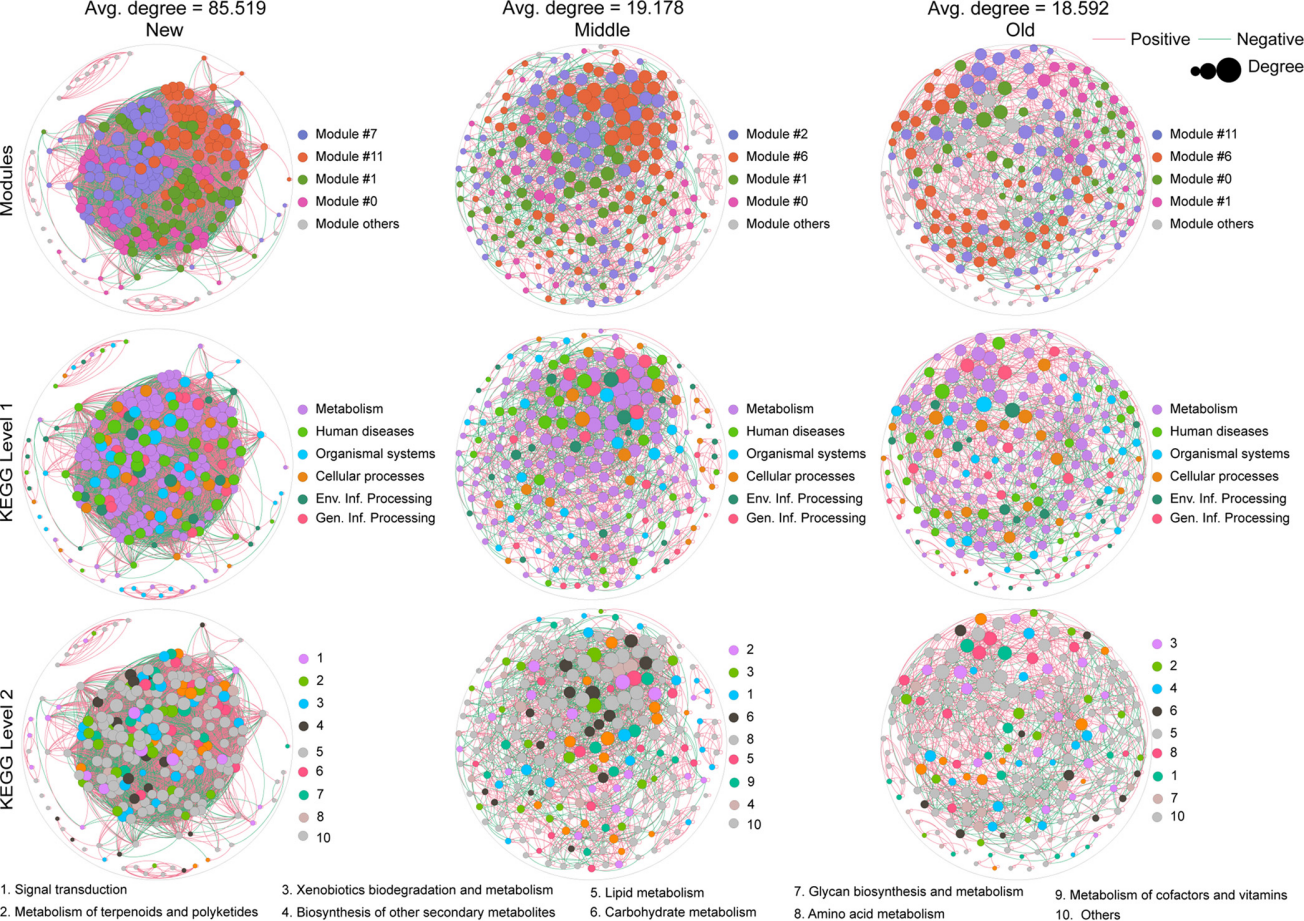
Variation in core soil microbial community functions associated with *C. oleifera* plantation ages. Functions are based on KEGG annotations. Red lines indicate positive correlations and green lines indicate negative correlations. The area of the node is proportional to the node degree, which is calculated from correlations of abundances for each KEGG pathway. Only correlations with an *r *of >0.6 or <−0.6 and a *P* value of <0.05 were included in the network.

Functional analysis of soil microbiomes was conducted by analyzing new (1-year), middle-aged (3- and 5-year), and old (7- and 9-year) plantation soil microbiomes. Co-occurrence network analysis based on annotations from the Kyoto Encyclopedia of Genes and Genomes (KEGG) indicated that new *C. oleifera* plantations exhibited the highest average degree of soil functional composition (85.519). The density of functional components in soil communities significantly decreased over time (with an average degree of 19.178 in new plantations), wherein the functional composition of old *C. oleifera* plantations was the least dense (average degree of 18.592) (Fig. 3 and Table 3). In the soil functional network for new *Camellia* plantations, four modules comprised more than 10% of the overall network, including modules 7 (30.19%), 11 (23.05%), 1 (21.75%), and 0 (15.58%). Among the three groups, node clustering degree was highest in the new plantations (with an average clustering coefficient of 0.669). The compositions of modules changed in the middle-age plantations, wherein modules 2 (33.19%), 6 (28.63%), 1 (21.75%), and 0 (15.58%) accounted for over 10% of the entire network. In particular, the first two modules were different than those in the new plantation soil networks (Fig. 3 and Table 3). Only the modules with the highest proportion changed compared to the intermediate-year networks (module 11, 32.5%) (Fig. 3 and Table 3).

**TABLE 3:** KEGG pathway co-occurrence network characteristics of *C. oleifera* planting years

Niche/yr	Node	Positive edge	Negative edge	Avg degree	Modularity^*a*^	Avg. clustering coefficient^*b*^	Avg. path distance^*c*^
New	308	8688	4482	85.519	0.964	0.669	1.872
Middle	241	1429	882	19.178	2.614	0.609	2.85
Old	240	1627	604	18.592	1.049	0.603	2.849

a Degree of nodes tending to differentiate into different network modules.

b Degree of nodes tending to cluster together.

c Network path distance is the length of the shortest path between two nodes within the network.

Analysis of KEGG level 1 annotations indicated that metabolism was the core functional category represented among the microbiomes of all three plantation age groups, while comparison of KEGG level 2 annotations revealed differences in core functions among soils of different-aged plantations. The functions within new plantation soil microbiomes primarily comprised the categories of signal transduction (7.14%), metabolism of terpenoids and polyketides (6.82%), xenobiotics biodegradation and metabolism (6.49%), biosynthesis of other PSMs (6.17%), lipid metabolism (5.19%), carbohydrate metabolism (4.87%), glycan biosynthesis and metabolism (4.87%), and amino acid metabolism (4.55%) (Fig. 3). In middle-aged plantation soils, xenobiotics biodegradation and metabolism (8.3%) and metabolism of terpenoids and polyketides (7.47%) became more prominent (Fig. 3). Lastly, xenobiotics biodegradation and metabolism (8.33%) and terpenoid and polyketide metabolism (7.08%) were similarly higher in old *C. oleifera* plantation soils. In addition, the biosynthesis of other secondary metabolites (6.25%) and carbohydrate metabolism (6.25%) gradually replaced the core functions of new *Camellia* plantation microbiomes and became core functional pathways in the soil microbiomes of old ones (Fig. 3).

**FIG 4:**
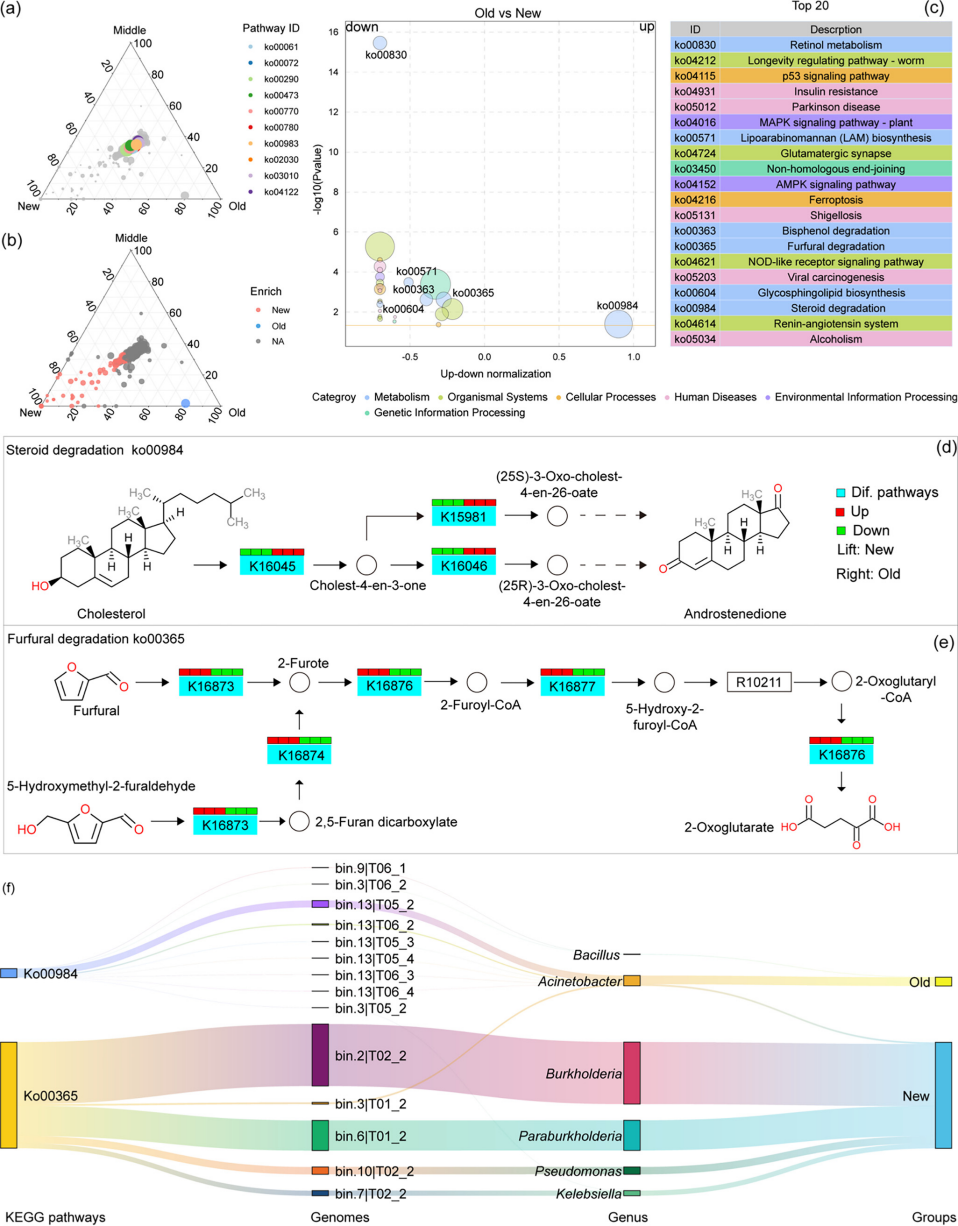
Effects of *C. oleifera* plantation age on degradation. (a) Variation in *C. oleifera* plantation ages with the distribution of the 10 most abundant KEGG pathways among soil metagenomes. (b) KEGG pathways significantly enriched in soil metagenomes from different plantation ages. (c) The 20 most abundant KEGG pathways that differentiated soil metagenomes from old and new *C. oleifera* forest plantations. (d) Differential enrichment of the steroid degradation pathway among soil metagenomes from different-aged plantations. (e) Differential enrichment of the furfural degradation pathway among soil metagenomes from different-aged plantations. (f) Genomes and genome annotation information for the pathways Ko00984 and Ko00365.

**FIG 5:**
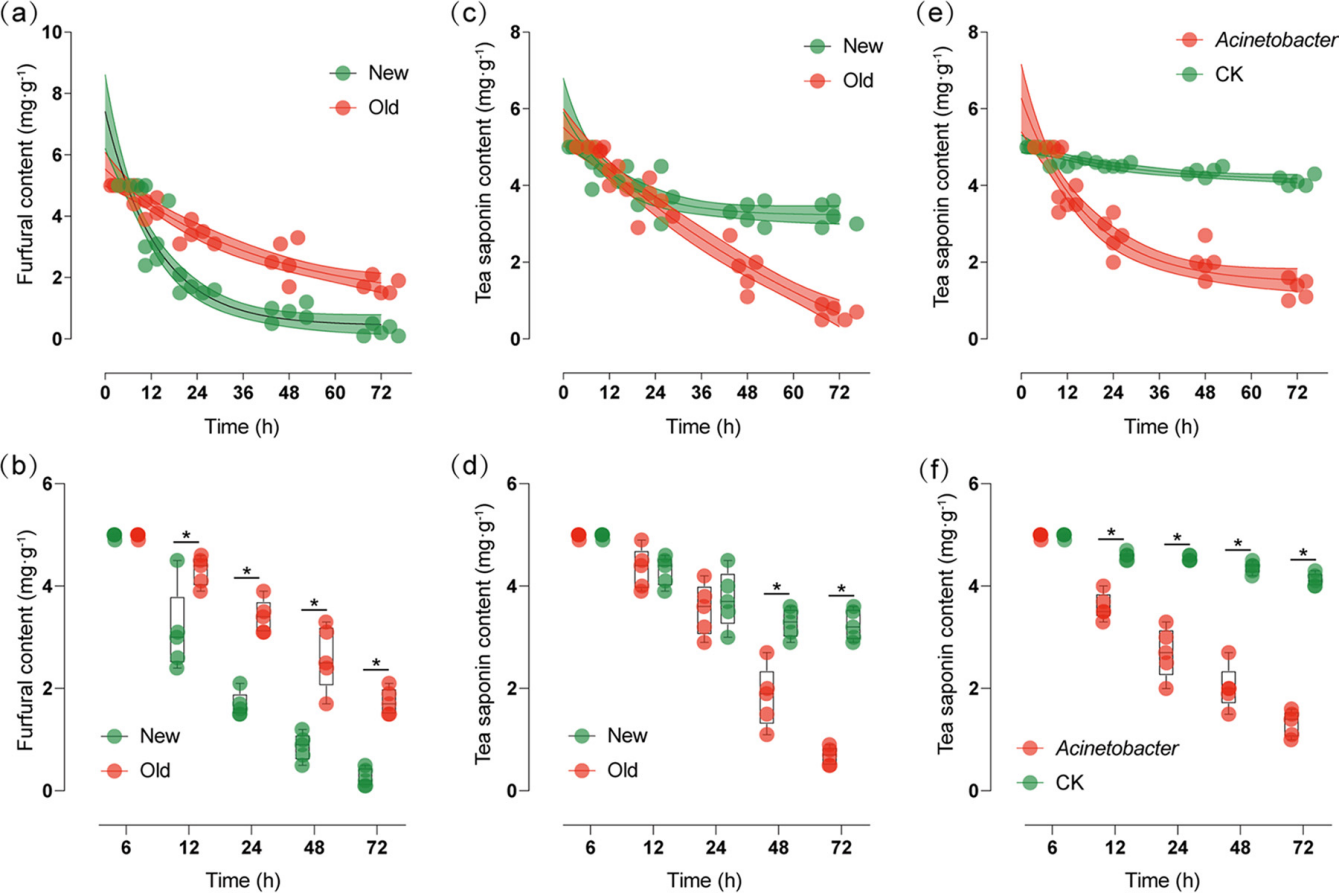
Degradation of furfural (a, b) and tea saponin (c, d) in soils of old and new *C. oleifera* plantations, and degradation of tea saponin (e, f) in sterile soil with *Acinetobacter* and control.

**FIG 6:**
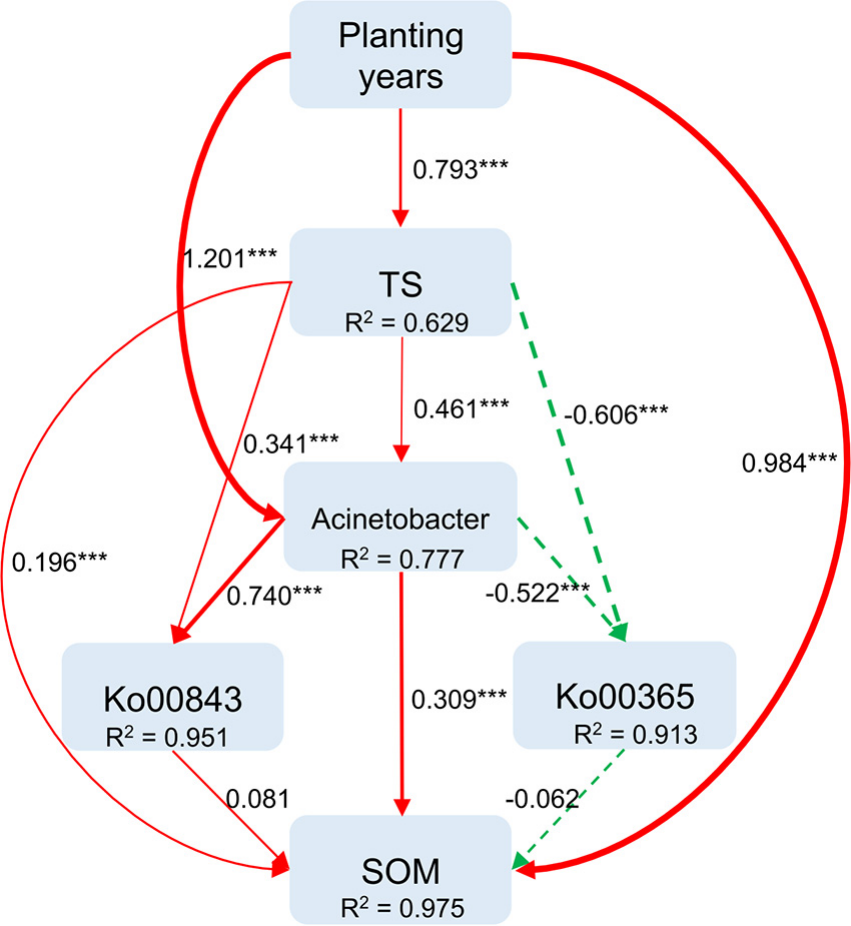
The structural equation model simulates the influence of PSMs on Ko00984 and Ko00365 pathways and SOM. (CFI = 0.992, GFI = 0.952, χ^2^/*df* = 3.862). Continuous or dashed lines indicate positive or negative relationships, respectively. Width of the arrow indicates strength of the effect. The digits besides the arrow are weight coefficients: *R*^2^, size of the variable; RMSEA, root mean square error of approximation. ***, *P* < 0.001.

To better understand the effects of plantation age on differences in soil microbiome function, KEGG pathway abundances were compared among the three different-age plantation soils. Differences were not observed among groups in the proportions of the 10 most abundant KEGG pathway groups. Overall, 71 pathways were significantly enriched in soil samples from new *Camellia* plantations, while 1 pathway was enriched in the old plantation soils, and no pathways were significantly enriched in the middle-age plantation soils (Fig. 4b and c). We further analyzed KEGG pathway differences between old and new *Camellia* plantation soils, observing the significant enrichment of the Ko00984 pathway (KEGG level 3: steroid degradation) in old plantation soils, while the other 19 pathways were enriched in new plantation soils, as described above (Fig. 4c). Ko00365 (KEGG level 3: furfural degradation) is an important pathway involved in cellulose degradation and was significantly enriched in new plantation soils, but was in low abundance in old plantation soils (Fig. 4e). Ko00984 is an important pathway involved in PSM degradation via the continuous decomposition of tea saponin into smaller-molecular-weight compounds (Fig. 4d). Genomic binning analysis was subsequently used to assemble genomes from the metagenomes of old and new *C. oleifera* plantation soils to better assess the context of the Ko00984 and Ko00365 pathways. A Sankey diagram visualization indicated that the Ko00984 pathway could be assigned to nine bacterial genomes, with two belonging to *Bacillus* and the other seven to Acinetobacter. In addition, the Ko00365 pathway was attributed to four bacterial genomes, belonging to *Burkholderia*, *Paraburkholderia*, Pseudomonas, and *Kelebsiella* (Fig. 4f). An SEM model showed that the spatial variation of SOM was influenced by planting years, tea saponin content, Acinetobacter abundance, Ko00984 and Ko00365; in total, 97.5% of SOM spatial variation was explained by the SEM model. All of these indices can directly affect the SOM: cultivated fixed number of years (β = 0.984), tea saponin content (β = 0.196), Acinetobacter abundance (β = 0.309), and Ko00984 can directly increase SOM (β = 0.081), while Ko00365 (β = −0.062) can directly reduce SOM. Planting years, tea saponin content and Acinetobacter abundance also have indirect effects on SOM. It is worth noting that planting years can indirectly affect SOM by affecting tea saponin content and Acinetobacter abundance; furthermore, Acinetobacter itself can indirectly affect SOM by affecting tea saponin content, Ko00984, or Ko00365 (Fig. 3–6).

### Functional verification of Acinetobacter activities.

Degradation experiments demonstrated that new *C. oleifera* plantation soils exhibited higher furfural degradation efficiencies than old soils (Fig. 5a). Further, furfural degradation was significantly different after 12 h of degradation experiments (*t* test: *t* = 3.02, *df *=* *8, *P* < 0.05) (Fig. 5b). However, the efficiency of tea saponin degradation was higher in old *C. oleifera* forest soils (Fig. 5c), although this degradation was relatively slow. Specifically, differences between treatment and control incubations were only significant after 48 h (*t* test: *t* = 4.85, *df *=* *8, *P* < 0.05) (Fig. 5d). Moreover, isolated Acinetobacter exhibited strong tea saponin degradation abilities (Fig. 5d). After 12 h of culture, the efficiency of tea saponin degradation in soils with Acinetobacter was significantly different from that of soils in the control group (CK; *t* test: *t* = 7.90, *df *=* *8, *P* < 0.05) (Fig. 5e).

## DISCUSSION

### Soil properties gradually deteriorated with increasing ages of *C. oleifera* plantations.

Density and water content are important indices of soil water storage capacity. Lower soil density represents greater soil porosity, resulting in better soil structure, performance, and water storage capacity (4). Older *C. oleifera* plantations had increased litter and canopy densities that could improve soil conditions and promote the formation of soft soils (Fig. 1c and Fig. S1). Nevertheless, the soil densities of old *C. oleifera* plantations were significantly higher than those of new ones, while the organic matter contents were also higher (Fig. 1c and Fig. S1). This indicated that litter decomposition was suppressed in old plantations, and the release of organic matter, nitrogen, phosphorus, and other substances into soils, as well as PSMs, was inhibited (3, 4, 23). Consequently, the total nitrogen and phosphorus in soil continuously drop, while PSM (i.e., tea saponin) accumulation keeps increasing significantly (Fig. 1c and Fig. S1). The lack of artificial supplementation means that the demand for N and P by plants is a primary reason for the significant decrease over time (Fig. 1c and Fig. S1). In contrast, the decomposition rate of litters with higher amounts of structural carbohydrates, such as tea saponin, was slower, although these PSMs may significantly accumulate within the soil as plantations age increases (3, 24).

### Concentration of tea saponin serves as the primary factor affecting soil microbiome structures.

Tea saponin is an important PSM of *C. oleifera* and belongs to the triterpenoid class of saponins (25). Its accumulation inhibits soil microbial decomposition in plant litter. This litter contains diverse PSMs, including alkaloids, phenolic compounds, and terpenes; all of these are often used against herbivores and pathogens, playing key roles in plant-microbe and plant-herbivore interactions (3). Tannins may slow rates of litter decomposition (26), and phenolic compounds can delay the colonization of litter by decomposer organisms (3, 27). Problematic root-derived phenolics in soils can also drive shifts in microbial community compositions and prime decomposition activities in forest soils (28). The increasing age of a plantation was associated with gradual decreases in soil microbiome diversity, and their community structures became simpler (Fig. 1a to c). Further, soil microbial communities exhibited similarities as plantation ages increased (Fig. 2) and tea saponin content was the primary factor driving this transition (Fig. 1c). Our results agree with those of other studies. Schütz et al. (29) disclosed that plant-derived PSMs and their derivatives may contribute to the regulation of plant microbiomes and their functional diversity; a considerable amount of allelopathic and microbiota-modifying metabolites is released from plant litter, thereby influencing soil microbial communities. Furthermore, benzoxazinoids, including microbial degradation products, have been long known to exhibit allelopathic properties in soils (30).

Tea saponin is also an important fungicide, composed of sugar chains, triterpenoids, and steroids or steroid alkaloids that are linked by carbon-oxygen bonds (21, 22). The structures of insect gut microbiomes have been significantly changed after feeding on fruits containing tea saponin (21). The soil microbiomes evaluated here also exhibited similar changes in response to saponin contents. Moreover, the relative abundances of Enterobacter, Acinetobacter, *Aquabacterium*, and *Rhodoplanes* were associated with the increasing tea saponin accumulation that accrued across plantation ages (Fig. 1e and f). In addition, Acinetobacter abundance was significantly and positively correlated with tea saponin accumulation (Fig. 1f). This bacterium is frequently associated with both aspen foliage and the gypsy moths that consume aspen foliage tissue, metabolizing phenolic glycosides within the foliage. Further, the species A. calcoaceticus and *A. oleivorans* were able to degrade catechin, modulating host physiology and metabolism to improve hexadecane utilization efficiency (31). Acinetobacter derived from wood-fed termite guts can efficiently degrade phenolic compounds by using phenol as its sole carbon source (32). Community interaction networks and random forest classification analyses conducted in this study indicated that Acinetobacter was one of the core soil bacteria in old *C. oleifera* plantations. The relative abundance of Acinetobacter was significantly and positively correlated with tea saponin accumulation, firmly suggesting that Acinetobacter is closely involved in tea saponin degradation.

### Accumulation of tea saponin affects soil decomposition functions.

Most plant species whose litter releases terpenoids, phenolics, steroids, and aliphatic acids generally inhibit litter decomposition and/or nutrient release (1, 33). Comparison of soil microbiome functional profiles from *C. oleifera* plantations of different ages at three sites revealed that metabolism is the primary KEGG level 1 function (Fig. 3). Correlation analysis and subsequent network analysis indicated that functional modules became less connected as plantation ages increased, while the predominant KEGG level 2 function changed among groups (Fig. 3). In the old-age plantation soils, functional modules were primarily annotated as xenobiotics biodegradation and metabolism, terpenoid and polyketide metabolism, and biosynthesis of other secondary metabolites (Fig. 3). As discussed above, tea saponin accumulation significantly altered soil microbial populations in the old *C. oleifera* plantation soils, which corresponded to changes in community functions. Pathways involved in nitrogen and phosphorus cycling, which are beneficial to soil quality improvement, were not significantly enriched. Similarly, a study of the impact of continuous crop planting on soil functions indicated that the pathways involved in nitrogen and phosphorus cycling were negatively affected, leading to crop yield reduction (34).

PSMs enter soils and significantly influence soil function following litter decomposition (24, 35–38). Our results indicated that a few KEGG pathways, primarily those involved in steroid degradation, were significantly enriched in the soils of old *C. oleifera* plantations with high tea saponin content (Fig. 4c and d). During the decomposition process, tea saponin is hydrolyzed into numerous steroids, glycosides, and other substances, and the enrichment of the Ko00984 pathway could be related to the degradation of tea saponin. Soil microbiomes may participate in the decomposition of tea saponin via enzymatic activities after tea saponin-derived PSMs enter soils (Fig. 4c and d). To verify this hypothesis, we used soils from old and new *C. oleifera* plantations to conduct tea saponin fermentation-degradation experiments, observing a much higher degradation efficiency in the old plantations compared to the new ones (Fig. 5c and d).

In addition, numerous pathways related to nutrient anabolism were significantly less abundant in the soil of old *C. oleifera* plantations; this was especially evident for the Ko00365 furfural degradation pathway, which is closely involved in cellulose degradation (Fig. 4c and e). These litters contain abundant cellulose which is initially degraded into hemicellulose, then furfural, eventually becoming an important component of SOM. Cellulose passes through soil bacteria via soil respiration, eventually being decomposed into carbon dioxide and water to complete carbon mineralization. In old *C. oleifera* plantation soils, the decomposition of furfural into organic acids was inhibited (Fig. 4c and e), probably due to the accumulation of SOM (Fig. S1a). Soils from old and new *C. oleifera* plantations were used in furfural decomposition experiments to assess this process. The results showed that furfural decomposition efficiencies in the soils of new *C. oleifera* plantations were higher than those in old soils (Fig. 5a and b), indicating that the accumulation of tea saponin and other PSMs in old *C. oleifera* plantations inhibited the increase of soil bacterial populations involved in cellulose degradation. This apparent inhibition of cellulose degradation pathways could be one of the factors underlying the continuous accumulation of SOM with increasing plantation age.

In addition, the Ko00984 pathway was primarily enriched in the genomes of Acinetobacter bacteria, while the Ko00365 pathway was primarily enriched in the genomes of bacteria involved in nutrient degradation (Fig. 4f). To confirm whether Acinetobacter can degrade tea saponin, an Acinetobacter strain was cultured from medium containing tea saponin as the sole carbon source, and its ability to degrade tea saponin was confirmed (Fig. 5e and f). The structural equation model also showed that planting years directly affected tea saponin accumulation, Acinetobacter abundance, and SOM, while tea saponin affected SOM through Acinetobacter abundance and the Ko00984 pathway (Fig. 5e and f; Fig. 6). Thus, the enrichment of Acinetobacter in the soil of old *C. oleifera* plantations is considered to be closely related to degradation of PSMs such as tea saponin (Fig. 5e and f; Fig. 6).

## CONCLUSIONS

In this study, the effects of *C. oleifera* plantation age on soil properties, microbiome structures, and microbiome functions were explored. Increasing plantation age led to significantly decreased quality in soil nutrient indices, while SOM and soil density increased dramatically. This could be the result of increased accumulation of tea saponin, an important PSM of *C. oleifera*. Tea saponin exhibits bactericidal toxicity, and its accumulation could be lethal to some soil bacteria involved in litter degradation. These dynamics would then affect pathways involved with SOM degradation, resulting in increased SOM. The homogenization of a soil microbiome weakens soil decomposition function, increases soil density, and causes soil compaction. Concomitantly, plant growth requires abundant nitrogen and phosphorus. Thus, as total nitrogen and phosphorus content in soil decreases over time, old *C. oleifera* plantation soil becomes barren and productivity drops remarkably. Therefore, correct measures should be taken for sustainable management of old *C. oleifera* plantations. First, functional bacterial fertilizers that can change soil microbial community structures should be applied in old *C. oleifera* plantations to accelerate litter decomposition. Second, the total nitrogen and phosphorus contents should be increased following a formula to enhance productivity. Furthermore, these measures should be taken together with the planting of selected understory plants in old *Camellia* plantations to achieve the goal of sustainable management.

## MATERIALS AND METHODS

### Study sites and sampling.

In this study, Qiangtian (28°11′51.61′′ N, 120°23′15.25′′ E), Quzhou (29°3′48′′ N, 118°36′15′′ E), and Jiande (29°01′ 32.06′′N, 119°37′ 28.45′′E) were selected as sample collection sites within Zhejiang, China. The sites exhibit a subtropical monsoon climate, and all are less than 300 m above sea level. Each site comprises a pure *C. oleifera* plantation where no chemical spray has taken place to control diseases, insects, and weeds. Medicinal plants, including *Dicranopteris pedata*, *Polygonum perfoliatum*, *Cynodon dactylon*, and *Imperata cylindrica* are also distributed throughout the sites. The annual average temperature there is 15 to 18°C, with the coldest in January (3 to 9°C) and the hottest in July (26 to 29°C).

Precipitation is abundant with an annual average of 1,600 mm. The clay content of the topsoil layer is 31.28% and the weathering degree of soil minerals is high, with a powdery clay ratio ranging between 0.83 and 0.98. The clay minerals in the soil are primarily kaolinite. The red soils are acidic, with a surface pH of <5.5.

At each site, soil samples were collected from a test plot where trees had been planted for 1, 3, 5, 7, and 9 years. Eighteen sampling points were randomly selected within plots for each planting year. Within a vertical ground projection of the canopy (1.5 m × 1.5 m), five points were randomly selected for sampling in June 2020. The top litter layer was removed from the soil, and a soil collector was wiped with the soil that was to be collected prior to sampling. The 0-to-20-cm soil layer was collected from each point and the soils collected for each age group plantation were randomly divided into six samples. A total of 90 soil samples were collected using the same method from three sites. Fresh soils were sorted by hand to remove roots and stones. All samples were further divided into two sets and placed in sterile bags. The first soil portion was immediately frozen in liquid nitrogen, stored on dry ice, and transported back to the laboratory, followed by storage at −80°C until subsequent DNA extraction. The other set was passed through a 2-mm sieve and stored at 4°C for determination of soil physicochemical properties.

### Soil physicochemical properties.

Soil physicochemical properties, including pH, moisture content (SW, %), density (SD, g·cm^−3^), organic matter (SOM, mg·g^−1^), total nitrogen (TN, mg·g^−1^), total phosphorus (TP, mg·g^−1^), available phosphorus (AP, mg·kg^−1^), total potassium (TK, mg·g^−1^), and available potassium (AK, mg·kg^−1^) were determined as described by Jiang et al. Tea saponin (TS, mg·g^−1^) contents were determined with liquid chromatography (21). Correlational analysis of all soil physicochemical properties and planting years was conducted using the R software package (v. 4.0) (21).

### DNA extraction and high-throughput sequencing.

Bacterial DNA was extracted using a Qiagen DNeasy blood and tissue kit, followed by determination of DNA concentrations and size distributions. The extracted DNA from each sample was used as input for library preparation using a TruSeq Nano DNA LT Library Prep kit. Prior to sequencing, an Agilent BioAnalyzer was used to evaluate library quality using an Agilent High Sensitivity DNA kit. After the libraries had been validated, they were quantified using a Quant-iT PicoGreen dsDNA assay kit (Promega). PCR was used to amplify the V5-to-V7 hypervariable regions of bacterial 16S rRNA genes. 16S rRNA gene amplification was performed using the forward primer 799F (5′-AACMGGAT-TAGATACCCKG-3′) and the reverse primer 1193R (5′-ACGTCATCCC-CACCTTCC-3′) (39). Paired-end sequencing (2 × 300 bp) of quality-validated samples was conducted on the Illumina Miseq platform using the MiSeq reagent kit V3 (600 cycles) (39). A target fragment size of 200 to 450 bp was used for library construction, which was performed at the Personal Biotechnology Company (Shanghai, China).

### Statistical analysis of diversity.

16S rRNA sequence quality filtering was conducted using Cutadapt (v. 1.9.1; https://cutadapt.readthedocs.io/en/stable/). Quality filtered paired-end reads were then merged using UCHIME (http://www.drive5.com/usearch/manual/uchime_algo.html) (40). The high-quality sequences were clustered into operational taxonomic units (OTUs) at the 97% nucleotide similarity level using UPARSE (40). Chimeras were identified and removed from the data set. DADA2 (41) was used to BLAST representatives of OTUs against the Silva database (https://www.arb-silva.de/) to obtain taxonomic information of each OTU (42). OTU subsampling was conducted to facilitate comparison among samples. Spearman correlation analysis was conducted for OTU abundances across samples, and only robust (Spearman’s *r* > 0.6 or *r* < −0.6) and statistically significant (*P* < 0.01) correlations were retained for network analysis. Network analysis of OTU abundances was conducted using the Psych software for R (43), followed by visualization with Gephi (44). Alpha diversity indices (Shannon diversity, Simpson diversity, ACE richness, Chao1 richness, and whole-tree PD) were calculated using QIIME2 (41). Beta diversity values (Bray-Curtis distances) were analyzed using a principal coordinates analysis (PCoA) (42). Statistical significance of bacterial community variation among the three regions was evaluated with an analysis of similarities (ANOSIM) test. To evaluate the most discriminatory taxa across samples, the relative abundances of bacterial taxa at the genus level were assessed using the random forest package (v. 4.6-14) for R with default parameters (45). Pairwise comparisons of environmental factors were conducted based on Spearman correlation coefficients that were calculated in R (46).

### Metagenomic analysis.

Metagenomic analysis was used to assess the functional changes of soil microbiomes based on respective forest settings (i.e., ages). PCoA analysis of microbiomes (described above) indicated that samples were segregated based on plantation ages, with three groups being evident (“new,” 1-year-old trees; “middle,” 3 and 5 years old; and “old,” 7 and 9 years old). Six soil samples from each group were randomly selected for metagenomic sequencing. Whole-genome shotgun (WGS) metagenomic sequencing was used to sequence total metagenomic DNA on the Illumina Novaseq/Hiseq high-throughput sequencing platforms using 150-bp paired-end sequencing after fragmenting the extracted soil DNA. An average of 12 Gbp per sample was generated from the sequencing libraries. Clean data were obtained by quality filtering the original data using Cutadapt (v. 1.17). Taxonomic annotation of sequence data was conducted using Kraken2, while Megahit was used for assembly (47). After assembly, contigs of  <200 bp length were removed (48). Species annotation of assembled contig sequences was integrated with the abundance tables for each sample to obtain species abundance tables at each taxonomic rank (i.e., domain, phylum, class, order, family, genus, and species). The MetaGeneMark software program (http://exon.gatech.edu/GeneMark/) (49) was used to identify prokaryotic open reading frame (ORF) and coding regions in addition to protein annotations. Nonredundant protein sequence sets were compared against the Kyoto Encyclopedia of Genes and Genomes (KEGG) database to annotate gene functions (50).

To better understand the role of planting years in the degradation of soil microbiome function, genome-resolved binning analysis was conducted to assess functions among individual populations. Spearman correlations were calculated among KEGG pathway abundances and only robust (Spearman’s *r* > 0.6 or *r* < −0.6) and statistically significant (*P* < 0.01) correlations were retained for network analysis. Network analyses of KEGG pathway abundances were performed using the Psych software for R (43) followed by visualization with Gephi (44) and Sankey diagrams (51).

### Soil function validation.

We specifically focused on the KEGG function Ko00984, which was significantly enriched in old *C. oleifera* plantation soils and is related to PSM degradation. To assess *in situ* functioning, we treated soils of old *C. oleifera* plantations with tea saponin (5 g · liter^−1^). After incubation for 24, 48, and 72 h, the remaining tea saponin contents were analyzed with high performance liquid chromatography (HPLC). To assess more explicitly the *in situ* functioning of Ko00365, which corresponds to a significantly suppressed carbon cycling pathway in soil microbiomes of *Camellia* plantations, we mixed furfural (5 g · liter^−1^), an intermediate product of cellulose degradation in the pathway, into the soils of old and new *Camellia* plantations. After incubation for 24, 48, and 72 h, residual furfural was measured using HPLC. To verify that the core bacterial populations were associated with tea saponin degradation, Acinetobacter were enriched using tea saponin as the single carbon source in media. Tea saponin (5 g · liter^−1^) as a substrate was then mixed with sterilized soil, after supplementing Acinetobacter cultures. In the control group (CK), Acinetobacter was replaced with sterile water. After incubation for 24, 48, and 72 h, residual tea saponin contents in the soils were measured with HPLC. Structural equation modeling (SEM) was used to gain a mechanistic understanding of the direct and indirect factors driving SOM across planting years. SEM analyses of SOM, planting years, TS, Acinetobacter, Ko00984, and Ko00365 were established as the main factors related to SOM accumulation. We selected the best model based on overall goodness of fit, including the chi-square (*χ*^2^) statistic, degrees of freedom (*df*), whole-model *P* value, goodness of fit index (GFI), and normed fit index (NFI). SEM analyses were conducted using Amos v.21.0 (IBM).

### Data availability.

Demultiplexed sequence data are available in the NCBI Sequence Read Archive (Bio-Project ID of soil microbiomes: PRJNA772371; Bio-Project ID of soil metagenome: PRJNA772552).
