# Supplementary figures and images for: Increased Tea Saponin Content Influences the Diversity and Function of Plantation Soil Microbiomes
Source: Microbiol Spectr. 2022 Jan 12;10(1):e02324-21. doi: 10.1128/spectrum.02324-21 (PMC8754145; doi:10.1128/spectrum.02324-21)

**Fig. S1.** Correlation between soil Physicochemical parameters and planting years.

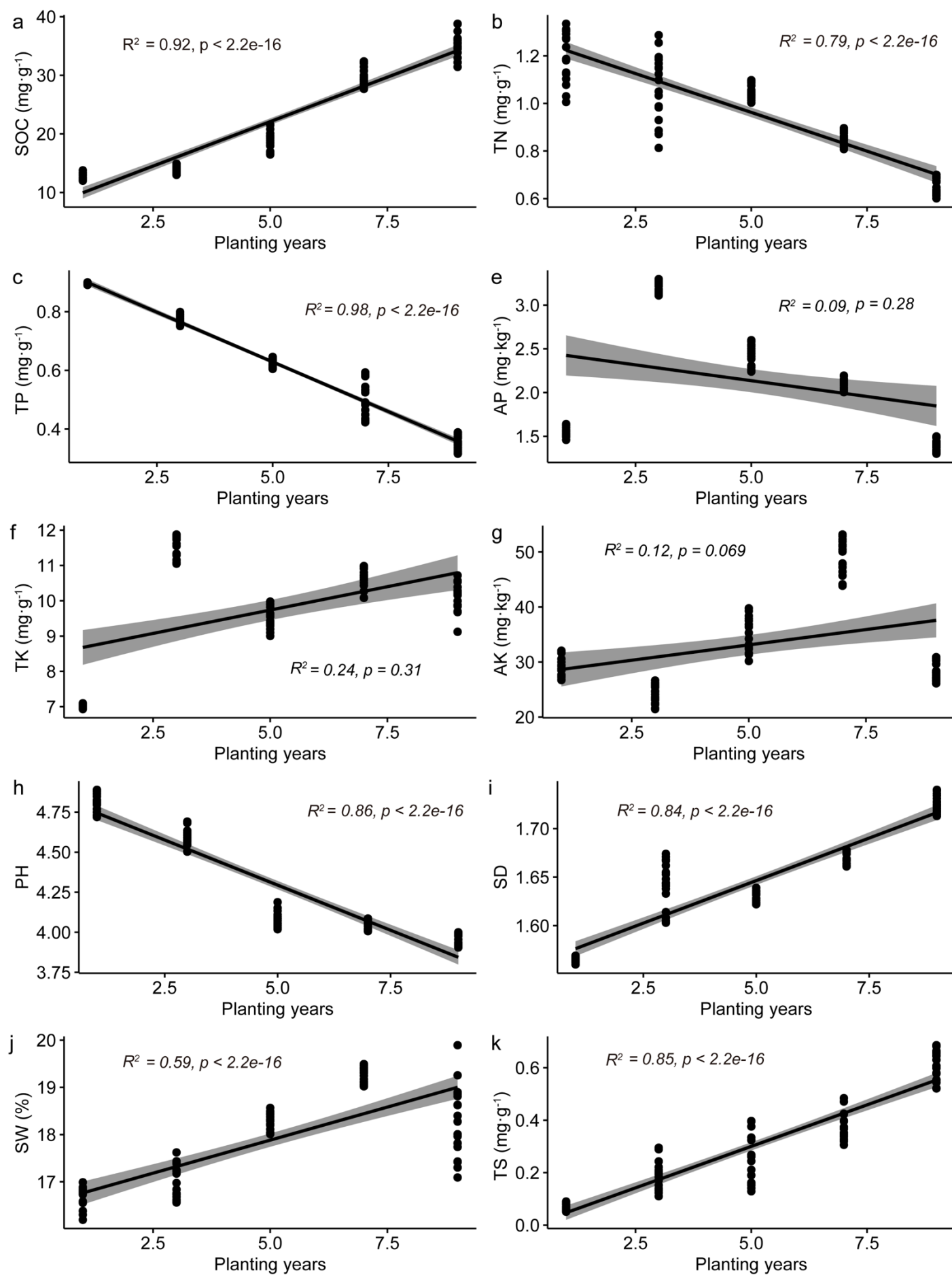

Supplement: SUPPLEMENTAL FILE 1 — Supplemental material. Download SPECTRUM02324-21_Supp_1_seq9.pdf, PDF file, 0.4 MB [file spectrum02324-21_supp_1_seq9.pdf]
